# Novel Approaches for Controlling and Analyzing Microorganisms in Foods

**DOI:** 10.3390/foods14193369

**Published:** 2025-09-29

**Authors:** Uelinton Manoel Pinto

**Affiliations:** Food Research Center (FoRC), Department of Food and Experimental Nutrition, Faculty of Pharmaceutical Sciences, University of Sao Paulo, Sao Paulo 05508-000, Brazil; uelintonpinto@usp.br; Tel.: +55-11-2648-0958

## 1. Introduction

Foodborne diseases continue to be a significant public health issue, despite improvements in food safety management systems, foodborne surveillance, better diagnostic tools, and better sanitation practices [1,2]. The World Health Organization (WHO) estimates that 1 in 10 people (~600 million individuals) fall ill due to the consumption of contaminated food every year, resulting in 420,000 deaths, of which 1/3 are children under 5 years old, and the loss of 33 million healthy life years [3]. There is also a huge economic loss, which is estimated at around USD 110 billion, mostly because of reduced productivity and healthcare costs. Additionally, contaminated or unsafe food contributes to trade barriers, financial losses, and increased food waste [3].

Food can become contaminated along the production chain, starting in the farm, but also during processing, distribution, preparation, and storage [4]. The complexity of the food supply chain, aligned with climate change issues, consumer demands for ready-to-eat and healthy foods, growing urbanization processes, emerging pathogens, and an increasing number of antibiotic-resistant organisms, has continuously raised new challenges and concerns for food safety authorities and the food industry across the globe [5,6]. It is also worth noting that the growth of vulnerable population groups, coupled with inadequate quality control and limited coverage of inspection services, and the intensification of large-scale food production, all contribute to the rapid transmission of pathogens, antibiotic-resistant organisms, and the occurrence of foodborne diseases [6].

Guaranteeing food safety and quality depends on a series of control measures and a deep understanding of microorganisms and their interactions within food ecosystems [4,7]. Understanding microbial ecology in foods is essential for developing preventive measures that can eliminate or minimize microbiological hazards as well as spoilage issues [8]. Fermentation and the use of microorganisms, particularly those with antimicrobial activity, play important roles [9]. Also important is the development of methods capable of detecting and monitoring pathogenic and spoilage organisms, as well as their toxins, to improve food safety and quality along the supply chain [1].

The development of new strategies for microbial control in foods that ensure safety while reducing chemical additives and preserving sensory and nutritional qualities is increasingly under consideration by the food industry and researchers worldwide, driven by consumer and regulatory demands [10,11]. Among these new alternatives, natural preservatives from plant, animal, or microbial origin have received considerable attention due to their antimicrobial and antioxidant properties [11,12]. Plant extracts, in particular, stand out for their alleged low cost, chemical diversity, and safety, with growing evidence of their ability to act not only as antimicrobials but also as quorum-sensing inhibitors, thereby modulating microbial communication and virulence [10,11,12,13].

## 2. An Overview of the Published Articles

This research topic aimed to gather original research and review articles on innovative strategies for the control and analysis of microorganisms in foods. There were five contributions to the topic, and this editorial will briefly introduce each of them, hoping to spark the curiosity of the reader to explore the full texts.

The study by Li and collaborators (Contribution 1) provides a compelling contribution to the ongoing search for alternative antimicrobial strategies by exploring how *Prunella vulgaris* L. extracts act against methicillin-resistant *Staphylococcus aureus* (MRSA). *P. vulgaris* is an edible–medicinal plant commonly used and found in Asia and Europe. The authors identified 28 phytochemicals via UPLC-ESI-MS/MS, including phenolic acids, flavonoids, and terpenoids, in addition to comparing the aqueous, ethanolic, and methanolic extracts. The results showed not only that the aqueous extract presented better activity, with lower MIC and MBC values, but also how these effects are achieved at the molecular and physiological levels by disrupting membrane and cell wall permeability, enzyme activity in both ATPase and Superoxide Dismutase, and metabolic disturbance by assessing the activity of the TCA cycle in MRSA. The work demonstrates additive interactions between the *P. vulgaricus* aqueous extracts with penicillin and erythromycin, with important implications for pharmacological use. The findings presented in the study strengthen the case for integrating medicinal-food plants into functional foods or even as adjunct therapies, as highlighted by the combinatorial use with antibiotics. However, further in vivo validation and safety evaluation should be assessed before these extracts can be applied in practical settings.

The research study of Mwangi, Shemesh, and Rodov (Contribution 1) aligned particularly well with the aims of the Special Issue by developing and validating a new phenolic-based green formulation combining the phenolic compound gallic acid (GA) with the Generally Recognized as Safe (GRAS) compounds hydrogen peroxide (H_2_O_2_) and lactic acid (LA). The dual (GA with H_2_O_2_) and triple formulations (GA with H_2_O_2_ and LA) were tested against several Gram-negative and Gram-positive bacteria commonly found in foods. The study demonstrated strong bactericidal synergistic effects (4-8 log CFU/mL reduction) for *Escherichia coli*, *Pseudomonas syringae*, *Pectobacterium brasiliense*, and *Bacillus subtilis* using the dual formulation, even though it had limited action against *L. innocua.* However, when adding lactic acid, the triple formulation allowed effective control of *Listeria innocua*, even though without increased reactive oxygen species (ROS) formation, as observed for the other bacteria. Interestingly, the authors show that *L. innocua*, under this triple formulation treatment, enters a viable but nonculturable (VBNC) state, characterized by maintenance of membrane integrity and ATP levels, despite significant loss of culturability. This phenomenon underscores that sanitizer efficacy must be assessed beyond just plate counts but rather by considering the physiological state of the cells. The study raised important questions about VBNC states and practical implications for food safety sanitization protocols.

On the theme of analyzing microorganisms in foods, Góngora and collaborators (Contribution 3) performed culture-dependent and metataxonomic sequencing approaches for the identification of microorganisms during coffee fermentation across 20 farms in Colombia. The authors characterized the taxonomy of both bacterial and fungal communities via 16S rRNA and ITS sequencing, showing how variables such as coffee variety, fermentation time, altitude, and physicochemical aspects of the mucilage and seeds shape microbial succession in the fermentation matrix, affecting sensory attributes, which were also evaluated by a trained sensory panel. The study links these microbial shifts to coffee quality attributes, including acidity, aroma, and fragrance, in addition to integrating culture-dependent counting and sensory analysis. The authors show that larger temperature variations, particularly peaks of high temperature, are associated with quality defects and with the growth of undesirable organisms such as *Citrobacter* and *Serratia*. Another parameter worth mentioning was the total acidity, in which the highest variations were associated with sensory defects, particularly due to the action of acetic acid bacteria (AAB). The metaxonomic analyses revealed that there was a core microbiome during coffee fermentation composed mainly of *Enterobacter,* lactic acid bacteria like *Leuconostoc* and *Lactiplantibacillus plantarum*, AAB, and yeasts of the *Hanseniaspora* genus. The findings of this study underscore the value of combining high-throughput microbial profiling with practical, quality-driven measures, directly contributing to improved processing practices.

The last two works of the research topic are review articles that deal with various food safety aspects. Contribution 4 from de Albuquerque and collaborators raises important concerns related to established ripening protocols and the safety of Brazilian Artisanal Cheeses (BAC). The authors point out that BAC have gained marketing value and prominence, particularly due to national and international recognition in festivals such as *Mondial du Fromage* in France and the World Cheese Awards in Wales, among others, as well as the recent advances in the legislation of these products in Brazil, which has allowed their commercialization across the country. The authors perform a rigorous examination of ripening protocols established by Brazilian regulatory bodies, drawing important remarks from studies that show the presence of different pathogens and indicator organisms in short-ripened raw-milk artisanal cheeses. By synthesizing evidence across 51 studies spanning different BAC production systems, they highlight critical gaps in current practices, including ripening times that are not sufficient to reduce hazardous bacteria for all cheese producers of particular cheese types. Additionally, they point out inadequate consideration of zoonotic pathogens like *Brucella* and *Coxiella,* which are not usually considered in most of the studies and may withstand the ripening conditions. Notably, the authors show that minimal ripening periods were defined for different types of artisanal cheeses in the country by several authors, but their study protocols were not standardized. Finally, the work underscores how environmental, milking, and herd health factors influence raw milk artisanal cheese safety and calls for standardized ripening assessment protocols when defining minimal ripening periods and conditions.

The final work in the Special Issue is a review article by Alves and collaborators (Contribution 5) on novel approaches to control biofilms in the meat industry. By surveying recent literature over the past decade, the authors clarify the ecology of multispecies biofilms in meat processing facilities, revealing how persistent background microbiota, including *Pseudomonas*, *Acinetobacter*, and psychrotolerant genera, may harbor and protect major foodborne pathogens. Their emphasis on biofilms in “hidden places” highlights how resistant to cleaning and sanitization these structures may be, reviewing vulnerabilities in current industrial practices but also demonstrating different control strategies. An important take-home message of the review is that, in addition to targeting recognized foodborne pathogens during cleaning and disinfection procedures, it is important to use multiple hurdles to dismantle recalcitrant biofilm structures and persistent environmental microorganisms. The work is organized in such a way that it provides a general overview on biofilm formation as well as microbial interactions within these structures, including the importance of quorum sensing signaling and inhibition strategies; the main pathogens that can be found in biofilms in the meat industry, including *Salmonella*, *L. monocytogenes*, *S. aureus*, and *Campylobacter*; the microbial ecology of biofilms in the meat industry, with an emphasis on culture independent DNA sequencing approaches; the hidden places with the potential to harbor biofilms; and control strategies, including eco-friendly methods.

## 3. Conclusions

The contributions to this Special Issue, “Novel Approaches for Controlling and Analyzing Microorganisms in Foods”, show the innovative strategies that have been developed to improve the control and analysis of microorganisms in foods. From natural antimicrobials from plant extracts and eco-friendly sanitizers to advanced approaches on the microbial profiling of fermented coffee beans and review articles exploring the safety of raw milk artisanal cheeses and the issues related to biofilms in the meat industry, these works collectively demonstrate the diversity of available tools that can be used to face persistent challenges and emerging food safety issues. Together, these studies reinforce the importance of integrating novel scientific insights with applied practices to ensure safer and more sustainable food systems.

## Data Availability

Not applicable.

## References

[1] Ávila Oliveira B.D., Gomes R.S., de Carvalho A.M., Lima E.M.F., Pinto U.M., da Cunha L.R. (2024). Revolutionizing food safety with electrochemical biosensors for rapid and portable pathogen detection. Braz. J. Microbiol..

[2] Nyachuba D.G. (2010). Foodborne illness: Is it on the rise?. Nutr. Rev..

[3] World Health Organization Food Safety. https://www.who.int/health-topics/food-safety.

[4] Pakdel M., Olsen A., Bar E.M.S. (2023). A review of food contaminants and their pathways within food processing facilities using open food processing equipment. J. Food Prot..

[5] Tchonkouang R.D., Onyeaka H., Nkoutchou H. (2024). Assessing the vulnerability of food supply chains to climate change-induced disruptions. Sci. Total Environ..

[6] Gargiulo A.H., Duarte S.G., Campos G.Z., Landgraf M., Franco B.D.G.M., Pinto U.M. (2022). Food safety issues related to eating in and eating out. Microorganisms.

[7] Ribeiro A.C., Almeida F.A.d., Medeiros M.M., Miranda B.R., Pinto U.M., Alves V.F. (2023). *Listeria monocytogenes*: An inconvenient hurdle for the dairy industry. Dairy.

[8] Auchtung J.M., Hallen-Adams H.E., Hutkins R. (2025). Microbial interactions and ecology in fermented food ecosystems. Nat. Rev. Microbiol..

[9] Graham A.E., Ledesma-Amaro R. (2023). The microbial food revolution. Nat. Commun..

[10] El-Saber Batiha G., Hussein D.E., Algammal A.M., George T.T., Jeandet P., Al-Snafi A.E., Tiwari A., Pagnossa J.P., Lima C.M., Thorat N.D. (2021). Application of natural antimicrobials in food preservation: Recent views. Food Control.

[11] Gyawali R., Ibrahim S.A. (2014). Natural products as antimicrobial agents. Food Control.

[12] Barbieri R., Coppo E., Marchese A., Daglia M., Sobarzo-Sánchez E., Nabavi S.F., Nabavi S.M. (2017). Phytochemicals for human disease: An update on plant-derived compounds antibacterial activity. Microbiol. Res..

[13] Machado I., Silva L.R., Giaouris E.D., Melo L.F., Simões M. (2020). Quorum sensing in food spoilage and natural-based strategies for its inhibition. Food Res. Int..

